# Empowering patients and educating staff – An online solution for the COVID era and beyond!

**DOI:** 10.1016/j.amsu.2021.102238

**Published:** 2021-04-01

**Authors:** M.A. Abdel-dayem, D.A. Brown, P.N. Haray

**Affiliations:** a Colorectal Surgery, Prince Charles Hospital, Merthyr Tydfil, Wales, UK; b Digimed®, UK; c University of South Wales, Wales, UK

**Keywords:** Online education, Colorectal, Patient empowerment

## Abstract

**Background:**

Bowel disease is a significant cause of significant morbidity and mortality around the world. Though colorectal cancer is a major cause for concern, there are a variety of other conditions which are chronic, debilitating and/or socially embarrassing. While the internet provides excellent resources, there is often conflicting and confusing material of doubtful veracity. There is pressing need for trainees and patients/carers to be able to access reliable resources whenever and wherever they are.

**Aim:**

To create an integrated, interactive platform providing reliable information on aspects of bowel disease for patients while addressing educational needs of surgical trainees and other healthcare professionals.

**Approach:**

Since 2006, we have progressed from leaflets, diagrammatic booklets to DVDs and then downloadable applications all of which, though very successful, had significant limitations.

Trainees struggle with balancing their educational needs with their service commitments. This online resource, www.colorectaleducation.com provides an opportunity to view detailed operative training videos on the go. The website also hosts detailed chapterised information videos for patients, care pathway videos and patient experiences. The modular design of the website allows for ease of updating and sequential expansion. The initial emphasis has been on colorectal cancer and the site is being gradually expanded to include a variety of other conditions.

**Results:**

The website gained widespread popularity with Google Analytics revealing steadily rising global hit rate with very low bounce rate for both sections. Structured feedback showed 96% satisfaction on both patient and professional sections.

**Conclusion:**

On-demand information became the norm with the use of smartphones/tablets. This website provides patients, surgical trainees and healthcare professionals access to information and education in clear reliable format, anywhere in the world. This is particularly relevant now as pandemic reduced opportunities for face to face patients consultations as well as for learners with educators.

## Highlights

• Colorectal diseases are common and there is always need for trainees and patients to be able to access reliable resources.
• Trainees struggle balancing educational needs with commitments. That’s obvious during pandemic rare training opportunities.
• Online training, conferences and resources proved to be effective during the pandemic. www.colorectaleducation.com provides information in reliable format to patients and healthcare professionals, anywhere in world.
• Structured feedback showed 96% satisfaction on both patient and professional sections.

## What does this paper add to the literature?

The paper presents an innovative solution of providing high quality education and support for patients as well as training for a range of staff, for a variety of serious colorectal conditions, incorporating state of the art technological developments AND with the potential for infinite expansion.

## 1 Introduction

Colorectal cancer is one of the most common cancers worldwide; it is the third most commonly diagnosed cancer in males and the second in females, with 1.8 million new cases and almost 861,000 deaths in 2018 according to the World Health Organization GLOBOCAN database. In addition, there is a wide range of other conditions, some with significant morbidity and others which cause a great deal of embarrassment and social stigma.

Improving health outcomes requires not only well educated and highly trained professionals but also patient empowerment with high-quality information and access to motivational material. Patient education is important for informed consent, reducing anxiety and improving treatment outcomes [1,2]. Integrated education enables inter-professional learning and collaborative patient care [3].

## 2 Aim

We aimed to create an integrated educational resource that would address the educational needs of professionals involved in managing colorectal disease while empowering patients and carers by providing reliable, consistent and high-quality information.

## 3 Methodology

***Design Remit:*** The resource we aimed for needed to be:

• easily accessible and available on demand
• consistent across all the sections, reliable, updatable and in a format easy to understand
• tailored to different audiences from specialist trainees in surgery and nursing to lay members of the public
• supportive, allay anxiety and provide motivational support, whilst maintaining its integrity

***Our Solution:*** A website advisory board was established to oversee the creation of an integrated educational website that allowed live streaming of audio-visual content, with representation from specialist colorectal surgeons, nurses, junior and senior surgical trainees, patients/carers, healthcare managers, informatics expertise as well digital medical media specialists (Digimed®).

Video content incorporated material filmed with patients, surgeons and healthcare professionals all with the appropriate consent. An approval process was established with various board members regularly collaborating on content relevant to their area of expertise**.**

The website, www.colorectaleducation.com, was launched in February 2018 with separate sections for patients and healthcare professionals. The patient section has been deliberately constructed to make it more easily navigable, bearing in mind that not all patients may be fully cognizant with web space browsing. The colorectal cancer area in the patient information section focusses on a virtual patient journey for colorectal cancer together with an enhanced recovery pathway. Patient experiences of dealing with colorectal cancer are well represented following feedback from the patient groups on the website advisory board.

**Fig. 1:**
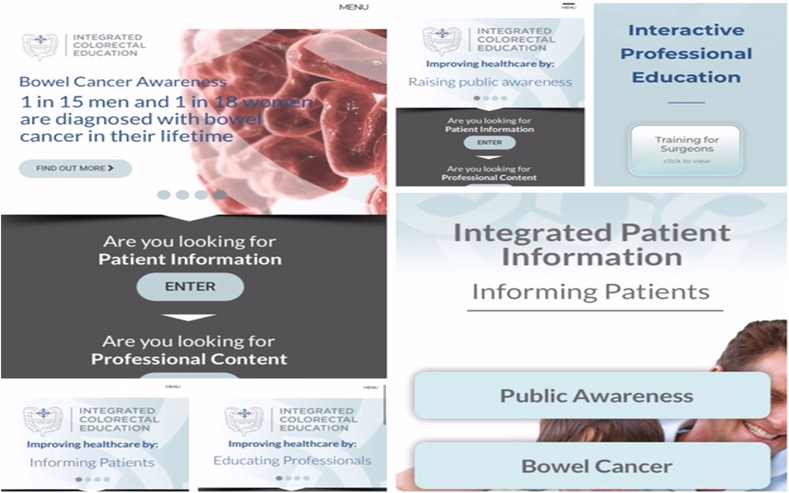
Sample pages from the website.

There is also an area providing some public education to raise awareness among asymptomatic members of the public as well as a downloadable interactive PDF to explain various standard colorectal operations with animated and annotated diagrams. This has proved particularly useful for patients as well as for medical and nursing students (Fig. 1).

The professional section, which can only be accessed once a disclaimer has been agreed (to prevent accidental exposure) contains specific areas for surgical training including detailed chapterised operative colorectal procedures as well as sections aimed at providing training for other allied health professionals including stoma nurses, ward and community staff.

Since its launch, we have continued to develop the site with sequential additions to existing areas as well as the creation of completely new sections such as hemorrhoid disease. We have been careful to produce parallel modules on patient information and staff education for all new topics, thus maintaining the ethos of this integrated education site. An ‘Ask the Experts’ interactive button is included for users to ask specific questions regarding any clinical or technical aspect of any of the videos.

The modular design of the website allows for ease of updating and sequential expansion. We have concentrated on colorectal cancer initially and are in the process of gradually including a wide range of benign conditions such as inflammatory bowel disease, diverticular disease, genetics, functional bowel problems and several proctological conditions.

The website is easily accessible on all types of smartphones, tablets or computers and is totally free of any charges for all users in both the patient and professional sections.

## 4 Results

The resource analytics reveal increasing popularity for both the patient and professional sections of the website and has been the first result on Google search for colorectal education. This is the result of pure organic growth rather than any promotional activity. Key data from Google Analytics for the website are as follows:

• 600 to 1300 new users each month with a steadily increasing trend over the past 12 months
• 28% of visitors return to the website
• 43% female and 57% male users
• The mean session duration of use is 4 min 20 s
• The Bounce Rate is very low at 2.5% (the percentage of visitors who leave after viewing one page, also defined as the percentage of single interaction visits)
• 49% of users start on the Patient side and 51% on the Professional side demonstrating the equal relevance of the website to patients and health professionals
• The sessions were accessed 81% via mobile phones, 18% via desktop, and 1% on tablets
• The reach is 56% UK, 15% Ireland, 12% USA, 3% Canada, 2% Netherlands, 2% India and 1% China and 9% shared between the rest of the world
• Of the first 100 feedback forms received, 96% of the users gave a high satisfaction rating for the website content, ease of use, accessibility of information and the value/relevance of the information provided

The website is regularly monitored, undergoing upgrade discussion and regularly audited by a website board which include a range of medical professionals, nursing and managerial representation, as well as patient and community representatives. The website board is chaired by a lay member of good standing in the community and meets on a regular basis to review comments and feedback, plan further development and approve content. The constant reviewing and updating of the contents is reflected in the fact that the site is currently in its ninth iteration.

In addition to providing static content, the website is also being used as a dynamic platform to deliver a range of webinars, courses and workshops accessible worldwide and aimed at a variety of healthcare professionals from medical students and nurses through to surgical trainees and established surgeons.

## 5 Discussion

Paper resources have long been the mainstay for patient education, but with limited usability [4] and to be of use, requires patients to have good vision, reading skills and the ability to understand the information provided [5].

Horwitz A. et al. published an interesting Scandinavian study in the Danish Doctors’ Weekly in 2009. In their study involving 111 patients, 21% never or rarely read patient information leaflets but relied on the doctor or the pharmacist. 62% of the interviewed had no problems reading or understanding the patient information leaflets. Among the 38% with reading problems, 57% had difficulties due to text-size, and 33% found the written language difficult to understand. 32% stated that they had stopped taking medication due to the information about adverse effects [5].

Although surgical textbooks have been around for centuries and still used in the mainstream, modern trainees have grown up in an era of digital technology and are used to different learning modalities and expect interactive and on-demand education. Plana NM et al., present evidence from a prospective, randomized, blinded trial involved 35 medical students supporting online digital simulation as a superior educational resource for novice learners, compared with traditional textbooks [6]. Their findings are supported by a study by Waltzman et al. [7].

The practice in our department was not dissimilar to other centers relying on paper leaflets for patient information. However, the staff were often left blind as to whether the patient had understood the information or even bothered to read the material at all. By the end of the last century, it was also becoming obvious that patients were accessing information from other resources including online. Colledge et al. reached similar conclusions in 2008, expressing that alternative resource to paper leaflets would be efficient in providing patient education [8].

DVDs emerged as the de facto medium to provide audio-visual information. A study from Germany emphasised DVDs as a resource to improve patient information [9]. This is true in our own experience, following the launch of our first DVD in 2009, specifically aimed at patients being diagnosed and treated with colorectal cancer [10]. This project won several national awards and generated considerable international interest and was the springboard for us to develop additional DVDs on Enhanced Recovery After Surgery (ERAS) and surgical training – The Stepwise Approach to Laparoscopic Colorectal Resections [11,12]. All of these products received positive feedback from patients and healthcare staff and improved patients’ level of education when it came to their journey and recovery [13].

A randomized control trial by Shariff et al. [14] evaluated the effectiveness of online multimedia in comparison to conventional “Study day” teaching in the acquisition of cognitive surgical skills. All trainees were assessed before and after the study period. The study concluded that online multimedia is an effective learning tool for cognitive skill acquisition in operative colorectal surgery and provides adjunctive training outside the operating room. The authors also observed that multimedia training resources are valuable and compliment traditional teaching methods and recommended that they should be developed for incorporation into post-graduate surgical training programs.

Smartphones have grown in popularity among the medical community [15] and there are now thought to be more than 7000 documented smartphone health applications (apps) in different medical specialties [16]although often blighted by sub-standard and outdated content resulting in questionable reliability [17]. The second and third authors’ experience includes four apps for patient and staff education which were designed and launched in 2014. Their experience with apps proved challenging. The proliferation of smartphones and tablets running across different platforms required different applications being commissioned and produced. Regular upgrades of each of the apps proved time consuming and expensive, especially as their aim had always been to keep all the products available free of charge.

Technological evolutions within the past few years have now made it possible to create reliable and high-quality educational resources which can be easily accessed on demand [18]. Our solution was an integrated educational website for colorectal surgery. Analytics have confirmed a steady global hit rate even without any search engine optimization or advertisements. This is in line with other educational sites, though strict comparisons are not possible due to the unique integrated structure of our educational website. The fact that more than a fifth of the visitors return is likely to be a reflection of the ease of use, navigability and content, as is the extremely favourable bounce rate of 9%. A study of a randomized selection of domains has suggested that the average website has a bounce rate of 49% with anything below 26% being considered to be a reflection of the excellence of the site [19]. Education allows patients to become more proactively involved in their management and therefore more compliant, producing successful outcomes [20] and is important not only for improving knowledge but also has positive psychological effects on patients [21]. Surgical trainees juggle a demanding workload alongside meeting stressful deadlines and therefore, educational resources should be easily accessible to aid their learning [22]. Our website has been getting a similar number of visits to the patient and the professional sections, thus confirming that we have got the content and the balance right.

This website's global reach, as reflected in the results above, can be taken as an affirmation of worldwide enthusiasm for such an informative integrated website. One of the challenges in designing such sites with a lot of audio-visual content is to make it user friendly across mobile devices with small screens. It is gratifying to note that nearly half the users have been able to access this site on such devices. As this is a ‘streamed’ service, the content is accessible on-demand wherever there is adequate bandwidth. The format has been carefully constructed to allow access across a wide range of operating systems and search engine browsers.

Patel et al. in their study involved 341 participants showing online resources contributed significantly to clinical activities with the majority of smartphone users willing to use their personal device [23]. The integrated nature of our educational resource empowers patients, allowing them to be proactively involved in the management of their conditions while simultaneously providing high-quality educational material for surgical trainees and other healthcare professionals. The streamlined modular design has allowed us to add updates/additions to existing material with relative ease. This has been exemplified by our ability to respond to the pandemic with relevant material in a timely fashion with the addition of the COVID section within the News heading as well as update our upcoming courses and events pages regularly.

Further development of this site will continue taking into account results from our ongoing detailed evaluation through direct user interviews, online feedback and other routes including relevant social media platforms. In the present climate with the pandemic, there is a paucity of opportunities for direct interaction for patients with clinicians as well as a lack of courses and traditional classroom teaching, making reliable information and education websites like this even more essential.

## 6 Conclusion

High-quality health care provision requires highly trained staff as well as well-informed patients. Our Integrated and interactive website, www.colorectaleducation.com provides a common platform for all those involved in colorectal surgery, to use, learn and reflect on. High quality, reliable colorectal education, on-demand and just a click away! Not restricted to operating theatre or hospital environment and free to all users. This interactive web-based resource is an integrated resource emphasizing the importance of providing support for patients as well as training for professionals, simultaneously. This is also a feature that makes this type of unique resources so successful.

## Declaration of competing interest

There are no conflicts of interest for any of the authors.

## Source of funding

No funding to declare.

## Provenance and peer review

Not commissioned, externally peer reviewed.

## Ethical approval

N/A.

## Consent

N/A.

## Trial registry number

1. Name of the registry:
2. Unique Identifying number or registration ID:
3. Hyperlink to your specific registration (must be publicly accessible and will be checked):

## Guarantor

Mahmoud Abdel-dayem.

## Author contribution

**Mahmoud Abdel-dayem:** Study design, data collection, data analysis, abstract and manuscript preparation, **David Brown: Digimed Medical Media.** Website design, material filming and uploading, **P.N. Haray:** Study design, project supervision, review of results and manuscript preparation.

## Originality statement

The Covid 19 pandemic has severely restricted opportunities for sharing supportive information with patients as well has impacted the provision of staff education and training. There is an urgent need for alternative modes of disseminating information to overcome these limitations and the online integrated educational resource presented here provides a reliable and globally popular alternative.
